# Research on the effect of charitable donations on regional medical level

**DOI:** 10.3389/fpubh.2024.1398649

**Published:** 2024-07-05

**Authors:** Wei Sun, Qilin Zhang

**Affiliations:** Center for Social Security Studies, School of Political Science and Public Administration, Wuhan University, Wuhan, Hubei, China

**Keywords:** charitable donations, regional medical level, Gini coefficient, provincial panel data, China

## Abstract

As the main vehicle for the tertiary distribution, charity has a certain regulating effect on regional medical level. However, the improvement of regional medical effect of charity has yet to be tested. Based on provincial panel data from 1997 to 2019, this study analyzes the impact of charitable donations on regional medical level. The empirical results show that charitable donations widen the gap of overall regional medical level in China, which not only results from the current period but also from charity accumulation in the past. The regional heterogeneity analysis show that charitable donations have expanded the regional medical level of the eastern and western regions, while have no significant effect on the regional medical level gap in the central region. The widening effect in the eastern region of charitable donations is the largest. In addition, charitable donations expand the regional medical level gap between urban and rural areas in China. Charity, as the regional medical development mechanism, has not yet played its due role and advantages in regulating regional medical level gap. Formulating and adjusting the corresponding charity promotion policies is necessary.

## 1 Introduction and literature review

Report to the 20th Communist Party of China National Congress states that the regional medical level gap is still wide, which is an important issue China is facing. According to the National Healthcare Administration, People’s Republic of China, the proportion of residents’ medical spending in total consumer spending will rise from 6.7 percent in 2002 to 8.6 percent in 2022. More than 70% of China’s residents have less than 70% of the reimbursement rate within the scope of the policy, and patients with serious diseases are more likely to use innovative drugs and other drugs outside the medical insurance catalog, and the personal burden is more heavy. According to the Statistics and Information Center of the China Health Commission, the total out-of-pocket rate of hospitalization expenses for patients with basic medical insurance was only 44.6 percent in 2018, and the out-of-pocket rate for rural patients was as high as 47.2 percent. Among hospitalized patients, 24.2% voluntarily left the hospital due to financial difficulties. However, due to the mutual offsetting dynamics of widening and narrowing the regional medical gap, the development medical gap of China has been hovering high for a period, which will be a basic trend of regional medical level in China (1).

Faced with serious income distribution situation in China, China government to improve the regional medical system and build a well-coordinated institutional framework, with a view to not only making the pie bigger but also improving the way the pie is divided up. As a major form of the current regional medical level gap in China (2), charity has become an important force in narrowing the gap between the high level of regional medical and the low level of regional medical, which is a great historical mission entrusted by the times.

After more than three decades of downturn, China’s philanthropy has grown considerably. The total amount of charitable donations has risen from 1.4 billion yuan in 1997^1^ (3) to 225.313 billion yuan in 2020 (4). Thus, what is the regional medical level of the rapidly growing philanthropy in China? Has it had an impact on narrowing the regional medical gap? And to what extent has it had an impact? This study intends to address the above issues to providing empirical evidence for the formulation and optimization of charity promotion policies.

Considering the complexity and versatility of the factors that affect regional medical level and how the effects are produced, factors that impact regional medical level need to be sorted out. Since Lewis (5) put forward the dual sector model, research on the causes of regional medical level gap has been advanced. Ranis and Fei (6) further revised and improved Lewis’ model, constructing a more complete theoretical system to explain the regional medical level gap. Beyond the 1980s, scholars mainly explained the factors of regional medical level gap from economic growth, technological progress, education, labor transfer, and economic globalization (7–9). Since the 1990s, Chinese scholars have also conducted many useful explorations on the causes of the regional medical level gap in China. Many scholars believe that China’s long-standing urban–rural dual economic structure (10) and the urban-biased institutional arrangements interacted with it (11, 12) are important factors contributing to the widening regional medical level gap in China. Zhan and Zhou (13) examined medical insurance reimbursement policy; total medical expenses, income level and aging society are the key factors that cause the gap in China’s medical development level. Their analysis showed that the impact of economic growth on the regional medical level gap is not equivalent between urban and rural areas. The education variable in public goods significantly widen the regional medical level gap, while institutional factors such as marketization reduce it. Other research has analyzed the impact of income of household (14), higher education policies (15), and social security (16) on the regional medical level of urban and rural residents.

At the current stage of China’s development, regional disparity has become a common problem (17–19). With the development of philanthropy, charity, especially charitable donations, as a gentle hand in regulating on regional medical level, has come to public attention along with the improvement of regional medical policies. In China, donation has an important impact on the development of medical treatment in various forms. For example, through the establishment of special projects, deep cooperation with enterprises and medical institutions, and a variety of relief agencies, these are important ways to realize the contribution of donations to medical development. At the theoretical and logical level, the idea that charity can reduce regional medical level gap is widely accepted. According to modern philanthropic economics, public goods theory and warm glow theory (see text footnote 1) are the two major theoretical cornerstones for explaining philanthropic behavior (20, 21), which provide philanthropic behavior with lasting motivation together with the goodness contained in traditional religions such as Buddhism, Christianity, and Taoism. Driven by this motivation, the role of charity in regulating regional medical level is also extremely unique. Firstly, it is a hybrid form of regional medical level that, depending on its source of funding, can have an impact on regional medical level^2^ in a way that no other form of distribution can (22–24), and the important role of charity in the improvement of regional medical level is widely recognized (25–28). Secondly, the mechanisms and paths of charitable donations in regulating regional medical level gap are characterized by directness, specificity, and complementarity. Charitable donations can directly regulate the gap between the rich and the poor through non-profit charitable organizations and transfer wealth between social groups with different levels of regional medical level (29, 30), which is a direct transmission mechanism that is difficult to achieve in other forms of distribution. And the earmarked characteristics of charitable donations ensure that donations are transferred to specific beneficiary groups. The function of government’s direct transfer payment in regional medical field is similar, but it is not so specific.^3^ Complementarity is characterized more by the fact that charity complements the regulating effects of regional medical level by taking personal emotional resonance and ethical and moral drives as its starting point (14, 31–33).

Due to the speculation and malfunction of charitable donations, its actual regional medical level gap have yet to be empirically tested. However, empirical research in this area is not very numerous and has not come to a consensus. Developed countries, represented by the United States, have more empirical research and more available data due to the earlier start of their philanthropy. Jackson (34) analyses the autonomous philanthropic institutions of the African Americans in Chicago during the period of rapid urbanization and segregation, which affirms the important role these institutions played in raising income levels and improving the quality of life for African Americans. Brest's (35) research on outcome-oriented philanthropy came to a similar conclusion, suggesting that this new type of philanthropy works well to help the poor and needy. Drawing on the constructing history of Ford Foundation and its recent goals, Soskis (36) explores whether the foundation can eliminate inequality, arguing that although there exists no definitive answer, it is better than doing nothing at all. On the contrary, some scholars believe that the development of charity will not lead to a narrowing gap of regional medical and may even produce some bad consequences. Reich (37) found that charity in the United States is not as well-developed as it appears to be. Contrarily, issues such as where, to whom, and how much charitable funds are invested can lead to potential fail of charity under the intervention of public policy, thereby exacerbating social inequality. The literature on the relationship between charity and regional medical level in China is relatively few, and most of them are normative studies with inconsistent views. At present, there is a big gap in the overall level of regional medical development due to the poor connection of relevant medical security systems in China, the need to standardize charitable medical behaviors, the lack of charitable mobilization capacity, and relevant institutional constraints. Most studies infer by logical deduction that the development of philanthropy in China can reduce the regional medical level gap to a certain extent (38–40). However, skeptics argue that charity does not necessarily reduce inequality, and that it may disregard the status quo of inequality or even be the cause of increased inequality (41, 42).

According to previous studies, there is a certain consensus on the significance and value of charitable donations in regulating regional medical level and narrowing the gap between the rich and the poor. However, empirical research on the effect of charitable donations on regional medical level is relatively weak, and the conclusions of these research are divergent. Therefore, research on this issue, especially empirical research based on Chinese data, needs to be strengthened. The possible marginal contributions of this paper are as follows: First, from the perspective of previous research, there is a certain consensus on the significance and value of charitable donations in standardizing regional medical standards and narrowing the gap between the rich and the poor. This paper studies the impact of charitable donations on regional medical standards, especially based on empirical data from China. Second, this paper uses a two-way fixed-effect panel model to analyze the impact of *per capita* charitable donations in each province on the regional medical level gap from 1997 to 2019, and conducts robustness test and heterogeneity analysis.

## 2 Data sources, variables, and model

### 2.1 Data sources

All raw data in this study are from the China Statistical Yearbook, the China Civil Affairs’ Statistical Yearbook, the statistical yearbooks of provinces, autonomous regions and municipalities from 1998 to 2020,^4^ and the statistical communiqué on economic and social development of provinces, autonomous regions and municipalities from 1997 to 2019.

### 2.2 Variables and descriptive statistics

#### 2.2.1 Explained variable

In this study, Gini coefficient (*Gini*) is chosen as the explained variable to measure the regional medical level gap. The measurement and calculation of Gini coefficient is very important and cumbersome, which needs to be explained in detail. The estimation of Gini coefficient in China, especially for the urban–rural decomposition of Gini coefficient under China’s dual economic structure has always been an important area of research, and many scholars have carried out a lot of exploration (43–45). However, no consensus has been reached. In addition, provincial data are more likely to be missing. Accordingly, provincial Gini coefficients are much less commonly measured in China. Using the non-equal grouping Gini coefficient, Chen (46), for the first time in a more complete way, calculated and analyzed the Gini coefficients for urban and rural residents in 21 provinces in China for the period 1995–2004. However, the urban and rural regional medical level data in China’s statistical yearbook have been grouped in different ways, such as interval grouping, quintile grouping, and non-equal groups, which causes the calculation caliber of the results to be inconsistent. In other literature estimating provincial Gini coefficient, only a single year is measured, or only a single and several provinces are observed, which is difficult to provide us with a comprehensive source of data and measurement methods (47). After comparing the literature on the measurement of provincial Gini coefficients, urban, rural, and the overall Gini coefficients of China’s 27 provinces from 1995 to 2010 tallied by Li and Zhao (48) are finally used. The use of area ratio formula can better solve the problem of inconsistent calculation caliber for Gini coefficient, while the data and time span coverage are more considerable. The specific calculations are as follows:


(1) $$\mathrm{Ginic}=1-\frac{1}{P_{c}W_{c}}\overset{n}{\underset{I=1}{\sum }}\left(w_{c\;i-1}+w_{c\;i}\right)\times P_{c\;i}$$

(2) $$\mathrm{Ginir}=1-\frac{1}{P_{r}W_{r}}\overset{n}{\underset{I=1}{\sum }}\left(w_{r\;i-1}+w_{r\;i}\right)\times P_{r\;i}$$

(3) $$\begin{array}{c} \mathrm{Gini}={RP}_{C}^{2}\frac{U_{C}}{U}\mathrm{Ginic}+{RP}_{r}^{2}\frac{U_{r}}{U}\mathrm{Ginir}\\ +{RP}_{c}{RP}_{r}\frac{U_{C}-U_{r}}{U} \end{array}$$

whereas equation (1) and (2) are formulas for calculating urban and rural Gini coefficients, respectively. P_C_, P_r_, W_ci_, and W_ri_ are the urban sample population, the rural sample population, the regional medical level of urban subgroup *i*, and the regional medical level of rural subgroup *i*, respectively. Equation (3) is the Sundrum formula for calculating the overall Gini coefficient for urban and rural areas,^5^ in which RP_C_, RP_r_, U_C_, U_r_, and U are the proportion of the urban population, the proportion of the rural population, regional medical level of the urban population *per capita*, regional medical level of rural population *per capita*, and the overall regional medical level *per capita*, respectively.

Further, considering the missing values in some provinces, data accessibility of other variables, and the calculation caliber, eight provinces out of 27 provinces^6^ are excluded. Finally, a total of 19 provinces (autonomous regions or municipalities) has been reserved, including Beijing, Hebei, Shanghai, Jiangsu, Zhejiang, Fujian, Guangdong, Shanxi, Jiangxi, Henan, Hubei, Inner Mongolia, Guangxi, Chongqing, Sichuan, Guizhou, Shaanxi, Gansu, and Xinjiang.

In addition, in the robustness tests section, this study employs the Theil’s index (*Theil*) as a substitute for the explained variables to measure the inequality of regional medical level (49). In the urban–rural heterogeneity section, the urban–rural regional medical level ratio (*incomeratio*) is introduced as an explained variable to measure the urban–rural regional medical level gap. The urban–rural regional medical level ratio is equal to the disposable regional medical level of urban residents *per capita* divided by the disposable net regional medical level of rural residents *per capita*.

#### 2.2.2 Explanatory variable

Charitable donations *per capita* (*pcharity*) is the core explanatory variable in this study. The data for 2010–2015 are calculated by the provincial social donations data from the China Statistical Yearbook divided by the population of the province in that year. Since the China Statistical Yearbook does not provide data by province before 2010 and after 2015, to ensure the consistency of the data,^7^ this study selected the sum of social donations (mainly reflecting donations received by the civil affairs departments) and the fund-raising regional medical level or added values of foundations, charitable associations, and social organizations by province in the current year (mainly reflecting donations received by charitable organizations) from the China Civil Affairs’ Statistical Yearbook, which is then divided by the population of that year. In the regression, the amount of charitable donations *per capita* are standardized to avoid the estimated coefficients being too small (*z pcharity*). In the robustness tests, the amount of charitable donations *per capita* received by the civil affairs department (*CApdonation*) is used as an alternative explanatory variable to re-estimate the results of the baseline model.

#### 2.2.3 Control variables

Based on previous research, the control variables are categorized into two groups. One is variables that measure the level of provincial philanthropic development, such as the number of foundations (*LNfoundation*). The other is provincial characteristic variables affecting regional medical level, such as the level of economic development (*LNpgdp*), which is deflated using the 1997 provincial GDP *per capita* as the base and then taken logarithms. The squared term of economic development (*LNpgdp2*) is also added to test the Kuznets hypothesis. The level of urbanization (*LNrcity*) is calculated by dividing the urban population by the population of the province in logarithmic terms. Since the population of long-term residents is not considered in China Statistical Yearbooks before 2004, the urbanization level is revised mainly referring to Zhou and Tian (50). The data for 2005–2019 are from the China Statistical Yearbook. The level of economic openness (*LNropen*) is obtained by converting the total amount of imports and exports of each province using the average USD to RMB exchange rate for the year, dividing it by GDP and then taking the logarithm. The level of financial development (*LNrfinance*) is calculated by dividing the loan balance of financial institutions in each province by GDP and taking the logarithm. Average years of education (*LNredu*) is calculated using the formula: (population of college degree and above education *16 + population of upper secondary education*12 + population of lower secondary education*9 + population of primary education*6)/total population aged 6 years and above. Endowment insurance coverage rate (*LNrss1*), health insurance coverage rate (*LNrss2*), and unemployment insurance coverage rate (*LNrss3*) are calculated as the ratio of the number of endowment insurance participants to the working-age population plus the older adult population, the ratio of the number of health insurance participants to the total population, and the ratio of the number of unemployment insurance participants to the working-age population, with the logarithms taken at the end, respectively.^8^

#### 2.2.4 Descriptive statistics

Table 1 reports the descriptive statistics for each variable. The mean value of Gini coefficient, which measures the disparity in regional medical level, is 0.38, very close to the Gini coefficient international alertness line level of 0.4. Its standard deviation is 0.05, which is relatively large, suggesting that income disparity fluctuates greatly between different provinces and years. Thiel’s index, alternative explained variable in the robustness test, has the same characteristics. The mean of explanatory variable, charitable donations *per capita*, is 31.73 yuan, which is relatively low. Charitable donations *per capita* in different provinces and years fluctuates largely, with the lowest charitable donation *per capita* amounting to only 0.14 yuan and the highest to 458 yuan. The charitable donations *per capita* received by civil affairs, which is the alternative explanatory variable in the robustness tests, has a mean value of 6.11 yuan, with the lowest being 0.04 yuan and the highest being 85.83 yuan, suggesting that the charitable donations *per capita* received by civil affairs also has the same characteristics. From the descriptive statistics of the variables, it can be concluded that the level of charitable development remained low and regional medical level disparity are evident during the sample period.

**Table 1:** Descriptive statistic of variables.

Variables	Mean	Median	Std. Dev.	Min.	Max.	Obs.
*Gini*	0.38	0.38	0.05	0.23	0.49	437
*Theil*	0.12	0.11	0.06	0.02	0.28	380
*pcharity*	31.73	6.41	64.95	0.14	458	437
*LNfoundation*	4.15	4.16	1.20	0.69	7.08	316
*CApdonation*	6.11	1.59	11.46	0.04	85.83	433
*LNpgdp*	11.11	11.29	1.63	7.70	14.03	437
*LNrcity*	3.84	3.85	0.34	3.13	4.50	437
*LNropen*	2.94	2.61	1.01	1.05	5.12	437
*LNrfinance*	3.57	3.50	0.68	2.15	5.26	437
*LNpedu*	2.12	2.13	0.14	1.69	2.54	437
*LNrss1*	3.36	3.38	0.88	1.34	4.63	437
*LNrss2*	2.91	3.52	1.92	−3.53	4.77	437
*LNrss3*	2.57	2.46	0.55	1.25	4.35	437
*Incomeratio*	2.75	2.68	0.55	1.60	4.59	431

### 2.3 Model

A two-way fixed-effects panel model is used to analyze the regional medical level gap of charitable donations, focusing on the impact of charitable donations *per capita* on regional medical level gap. The baseline model of this study is set as follows:


(4) $${\mathrm{Gini}}_{\mathrm{it}}=\beta_{0}+\beta_{1}{\mathrm{pcharity}}_{\mathrm{it}}+\beta_{2}X_{\mathrm{it}}+u_{i}+year_{t}+e_{\mathrm{it}}$$

whereas *Gini* is the Gini coefficient for each province, which measures the regional medical level gap across provinces. *pcharity* is charitable donations *per capita* in each province, which indicates the level of charitable donation in each province. *X* is a series of control variables. One group is variables measuring the level of philanthropic development, calculated by the number of foundations in each province. The other group is provincial characteristic variables affecting the regional medical level gap, including GDP *per capita*, the proportion of urban population, the share of total imports and exports, the percentage of loan balances of financial institutions, average years of education *per capita*, and the coverage rate of endowment insurance, medical insurance, and unemployment insurance. *i* denotes province; *t* denotes year; *u_i_* is a province fixed effect; *year_t_* is a year fixed effect; *e_it_* is the error term. *β_1_* in equation (4) is the core coefficient of this study. *β_1_* greater than 0 indicates that the charitable donations *per capita* has a positive effect on provincial regional medical level gap (i.e., charitable donations *per capita* widens the provincial regional medical level gap). Conversely, charitable donations *per capita* narrows the provincial regional medical level gap.

## 3 Empirical results and analysis

### 3.1 Benchmark regression

Firstly, the applicability of random effects and the fixed effects model are analyzed using the Hausman Test, which rejects the null hypothesis at 1% significance level, implying that there is a correlation between the unobservable fixed effects and other variables in the model. Thus, the fixed effects model is chosen. The results of the two-way fixed effects model are reported in Table 2, where column (1) shows the result of only adding the number of foundations, which measures the level of philanthropic development. Column (2) is the results only adding provincial characteristic variables. Column (3) is the result of adding both the number of foundations and the provincial characteristic variables. As the effect of charitable donations *per capita* on the regional medical level may not necessarily come exclusively from the current period, column (4) shows the result of adding one-period lagged term of the core explanatory variable on top of column (3).

**Table 2:** The impact of *per capita* charitable giving on income distribution.

Variables	Explained variable: gini coefficient			
	(1)	(2)	(3)	(4)
*z pcharity*	0.004^***^ (3.03)	0.007^***^ (3.87)	0.011^***^ (5.43)	0.009^***^ (3.33)
*LNfoundation*	−0.010^***^ (−3.05)		−0.007^**^ (−1.98)	−0.007^**^ (−2.03)
*Lz pcharity*				0.003 (1.10)
		
*LNpgdp*		0.031 (1.37)	−0.047 (−1.39)	−0.048 (−1.41)

*LNpgdp^2^*		−0.001 (−1.63)	0.002 (1.62)	0.002 (1.63)

*LNrcity*		0.039^**^ (1.98)	0.110^***^ (3.03)	0.104^***^ (2.90)

*LNropen*		−0.015^***^ (−4.34)	−0.009^**^ (−2.34)	−0.009^**^ (−2.38)

*LNrfinance*		0.014^*^ (1.85)	0.030^***^ (3.02)	0.029^***^ (2.95)

*LNpedu*		−0.022 (−0.52)	−0.072 (−1.54)	−0.069 (−1.48)

*LNrss1*		−0.004 (−0.71)	−0.007 (−1.06)	−0.007 (−1.02)

*LNrss2*		0 (−0.10)	0.005 (1.44)	0.004 (1.31)

*LNrss3*		−0.017^***^ (−2.71)	−0.029^***^ (−3.49)	−0.028^***^ (−3.41)

*cons*	0.436^***^ (37.80)	0.103 (0.71)	0.419^**^ (2.03)	0.397^*^ (1.91)
*Province FE*	Y	Y	Y	Y
*Year FE*	Y	Y	Y	Y
*N*	316	437	316	316
*Adj R^2^*	0.548	0.624	0.601	0.602

The *t* values are given in parentheses. ^*^, ^**^, and ^***^ represent significance levels of 10, 5, and 1%, respectively.

Table 2 shows that the estimated coefficients of charitable donations in four columns are all positive and significant at the 1% level, indicating that charitable donations *per capita* widens the regional medical level gap. Column (3) shows that the Gini coefficient improves by 0.011 for each standard deviation increase in charitable donations *per capita* (the core explanatory variables are standardized) after controlling other variables. The addition of a one-period lagged term of explanatory variable in column (4) increases the Gini coefficient by 0.009 for each standard deviation increase in charitable donations *per capita*, which is smaller than the estimated coefficient in column (3), suggesting that the accumulation of charitable donations in previous period has some impact on the regional medical level in the current period.

The results of the benchmark regression may be due to the following reasons: Firstly, in terms of the receipt of charitable resources. Although the volume of charitable donations in China is growing rapidly, the absolute amount of charitable donations *per capita* is still small, and the resource mobilization capacity of the charitable sector is still very limited. National charitable donations totaled 137.974 billion yuan in 2019 (51), merely 0.14% of the national GDP^9^ (52), compared to 2.10% in the United States^10^ (53, 54). According to the World Giving Index 2021 published by the United Kingdom Charities Aid Foundation in 2021, China ranked 95th in the Giving Index among 114 countries surveyed in 2020, and 85th in charitable donations (55). Low levels of charity donations, especially the level *per capita*,^11^ have led to a weak impact of charitable donations on regional medical level gap. Secondly, in terms of the allocation of charitable resources. The allocation of charitable resources will affect its regulating effect. In recent years, there have been frequent charity scandals, mismatches and inefficient allocation of charitable funds and goods, which have caused adverse social impacts, dampened public enthusiasm for donations, and further weakened the regulating effect of charity. Thirdly, in terms of the tools used to incentivize charitable donations. Tax benefits for charitable donations are used by governments to incentivize social giving, which are also highly controversial. Critics argue that tax benefits for donations are essentially a government’s subsidy to donors, with higher level of regional medical benefiting more than lower level of regional medical. Thus, tax benefits are therefore an inverted subsidy that increases inequality (56, 57). Accordingly, charitable donations are likely to widen the regional medical level gap and offset the regulating effect of charitable donations on regional medical level gap.

In terms of variables measuring the level of philanthropic development, columns (1), (3), and (4) show that the number of foundations reduces the regional medical level gap. Column (4) shows that 1 unit increase in the logarithm of the number of foundations reduces the regional medical level gap by 0.007 unit, which is significant at the 5% level. The main reason is that, with the guidance and support of policies, the rapid growth of corporate and community foundations and the consequent expansion of the scale of endowments and charitable assets will significantly narrow the regional medical level (58).

In terms of control variables: (1) The estimated coefficient of the level of urbanization (*LNrcity*) is significantly positive, indicating that urbanization has widened inequality in regional medical level, mainly due to the fact that China’s traditional path of urbanization paid too little attention to social equity issues and inequities in education, healthcare, and older adult care services under the urban–rural hukou system, which should be improved with the process of urbanization toward the right side of the inverted U-shaped curve (59). (2) The estimated coefficient of the level of financial development (*LNrfinance*) is significantly positive, indicating that financial development has widened the regional medical level gap. An important reason is that there are certain cost thresholds and credit constraints in accessing financial market and enjoying financial services in China. The affluent class, due to its accumulated wealth and good reputation, can enjoy more financial services than the poor class, thereby obtaining higher investment returns, which widens the gap between the rich and the poor (60). Thus, it is recommended to vigorously promote the development of inclusive financing (61). (3) The estimated coefficient of the level of economic openness (*LNropen*) is significantly negative, suggesting that trade openness and economic globalization contribute to reducing inequality in regional medical level. Previous research has indicated that trade globalization will exacerbate regional medical level gap in the short run but narrow it in the long run (62). Therefore, China should participate in the process of economic globalization in a more active role, expand the degree of economic openness, and improve the income disparity. (4) The estimated coefficient of unemployment insurance (*LNrss3*) is significantly negative, indicating that the development of unemployment insurance reduces the regional medical level gap. Thus, the coverage rate of unemployment insurance should be expanded by paying attention to the low-income groups with unstable employment status and high unemployment rate, thereby gradually including them in the insurance coverage (63). Other control variables, including the level of economic development, average years of education, and endowment and health insurance coverage, have no significant effect on the regional medical level gap.

### 3.2 Robustness tests

#### 3.2.1 Using the sub-period of 2008–2019

After the Wenchuan Earthquake in 2008, people’s enthusiasm for charity soared in China. Thus, 2008 is also called the first year of China’s philanthropy, after which China’s public welfare and charity began to develop in specialized, organized, and synergic direction. Therefore, the development of charity after 2008 is different from that before 2008. The sample for 2008 and later is remained and the regression is conducted again to verify the robustness of the benchmark results, which is shown in columns (1) and (2) of Table 3. Column (2) reports the results of adding one-period lagged term of the explanatory variable.

**Table 3:** Robustness tests.

Variables	Gini coefficient				Theil’s index	
	Using sub-period after 2008		Replacing explanatory variables		Replacing explained variables	
	(1)	(2)	(3)	(4)	(5)	(6)
*z pcharity*	0.007^***^ (2.97)	0.009^***^ (4.21)			0.005^***^ (3.36)	0.008^***^ (7.20)
*Lz pcharity*		0.002 (0.79)				0.005^***^ (3.17)
*z CApdonation*			0.006^***^ (4.16)	0.007^***^ (4.88)		
*Lz CApdonation*				0.004^***^ (2.65)		
*Other control variables*	Y	Y	Y	Y	Y	Y
*Province FE*	Y	Y	Y	Y	Y	Y
*Year FE*	Y	Y	Y	Y	Y	Y
*N*	225	225	308	312	316	316
*Adj R^2^*	0.604	0.605	0.597	0.592	0.916	0.913

The *t* values are given in parentheses. ^*^, ^**^, and ^***^ represent significance levels of 10, 5, and 1%, respectively.

#### 3.2.2 Replacing explanatory variables

The core explanatory variable of this study, charitable donations *per capita*, comes from the sum of charitable donations received by civil affairs, foundations, charitable associations and social organizations. The core explanatory variable is replaced with the amount of charitable donations *per capita* received by civil affairs (*CApdonation*) to verify the robustness of the benchmark results, which is shown in columns (3) and (4) of Table 3. Column (4) reports the results of adding one-period lagged term of the explanatory variable.

#### 3.2.3 Replacing explained variables

The explained variable is replaced with Theil’s index (*Theil*) to verify the robustness of the results, which is shown in columns (5) and (6) of Table 3. Column (6) also reports the results of adding one-period lagged term of the explanatory variable.

Table 3 reports the results of robustness tests. After using the sub-period and retaining data only from 2008 and later years for the regression, the basic findings are consistent with the benchmark regression. Charitable donations *per capita* widens the regional medical level gap, with the Gini coefficient increasing by 0.009 for each standard deviation increase in charitable donations *per capita*, which is significant at the 1% level [column (4)]. After replacing the core explanatory variables with the amount of charitable donations *per capita* received by the civil affairs, the basic findings are consistent with the benchmark regression. The amount of charitable donations *per capita* received by the civil affairs sector widens the regional medical level gap, and a one standard deviation increase in the amount of charitable donations *per capita* received by the civil affairs sector increases the Gini coefficient by 0.007, which is significant at the 1% level [column (4)]. After replacing the explained variable with Theil’s index, the basic findings are still consistent with the benchmark regression. The amount of charitable donations *per capita* widens the regional medical level gap, and a one standard deviation increase in charitable donations *per capita* is associated with a 0.008 increase in the Theil’s Index, which is significant at the 1% level [column (6)]. Therefore, the benchmark regression results are robust.

#### 3.2.4 Endogenous test

Considering the possible endogeneity problem between charitable donations and regional medical level, the 2SLS method is used to test, referring to the study of Gu and Ouyang (64), which adopts charitable donations lagged by one period [pcharity(*t*−1)] as the instrumental variable (IV), and the results are shown in Table 4. The first-stage estimation results show that the IV has a better explanatory power for the endogenous variables. The Kleibergen-Paaprk LM test rejects the original hypothesis of under-identification of IV, while the Kleibergen-Paap rk Wald *F* statistic is significantly larger than the critical value of the Stock-Yogo in the test of weak identification of IV. The second-stage estimation results show that the estimated coefficient of charitable giving on regional healthcare levels remains significantly positive after accounting for endogeneity, further corroborating the findings of the benchmark regression.

**Table 4:** Endogenous test.

Variables	The first stage	The second stage
*IV*	0.003^***^ (4.21)	
*pcharity*		0.008^***^ (7.20)
*Kleibergen-Paaprk LM*	45.426^***^	
*Kleibergen-Paaprk Wald F*	62.952 {16.38}	

*Other control variables*	Y	Y
*Province FE*	Y	Y
*Year FE*	Y	Y
*N*	297	297
*Adj R^2^*	0.605	0.592

Figure in {} is the Stock-Yogo test thresholds.

### 3.3 Heterogeneity analysis

#### 3.3.1 Regional heterogeneity

Considering China’s traditional division standards of three major regional of East, Central and West, the impact of charitable donations *per capita* on regional medical level is examined.^12^ Consistent with the previous section, Hausman test is used to reject the random effects model. Thus, fixed effects model is chosen. Table 4 reports the basic results for different regions, as well as the results of adding one-period lagged term of the explanatory variable.

Table 4 shows that the East [Columns (1) and (2)] and the West [Column (6)] are consistent with the nation (i.e., the amount of charitable donations *per capita* significantly widens income distribution gap). Column (2) and (6) indicate that after the addition of one-period lagged term, Gini coefficient in eastern region increases by 0.013 for each standard deviation increase in the amount of charitable donations *per capita*, which is significant at the 1% level. Column (6) shows that after the addition of one-period lagged term, Gini coefficient in the western region increases by 0.006 for each standard deviation increase in charitable donations *per capita*, which is significant at the 5% level. Although charitable donations *per capita* expand the regional medical level gap in both regions, the estimated coefficient in the eastern region is larger than that of western region. Although the coefficients in the central region are not significant, they are also positive and are smaller than the estimated coefficient in the eastern and western regions, which indicates that charitable donations *per capita* to expand the regional medical level gap in the eastern region has the largest effect.

Explanation for the results above can be made as follows. Currently, most of China’s charitable donations come from the affluent class. According to relevant statistics, the top 100 enterprises donators in 2018 accounted for 34.8% of the overall donations, while the top 100 individual donators accounted for 29.2%, which accounted for 64% of the total together. Other donators only account for 36%, indicating that the head donators are the main force of social donations (51). The level of economic development in the eastern region is superior to that of the central and western regions, and most of the enterprises and individuals who make the most donations are also clustered in the eastern region. According to the theory of elite philanthropy, although the affluent class makes large donations, they receive more substantial returns, of which scale is staggering (65, 66). Thus, while elite philanthropy ostensibly boasts of fairness, justice and giving back to community (67, 68), their charitable donations do not play a role in narrowing regional medical level gap, which widens inequality contrarily (69, 70). Therefore, the widening effect of charitable donations *per capita* on regional medical level is more prominent in the eastern region. Therefore, the widening effect of charitable donations *per capita* on regional medical level is more pronounced in the Eastern region of China.

#### 3.3.2 Urban–rural heterogeneity

The urban–rural regional medical level ratio is used as an explained variable to examine the effect of charitable donations *per capita* on the urban–rural regional medical level gap. Similar to the previous section, Hausman test is used and fixed effects model is chosen finally. Table 5 reports the results of urban–rural heterogeneity. Columns (2) reports the regression results by adding a one-period lagged term of explanatory variable.

**Table 5:** Regional heterogeneity.

Variables	Explained variable: Gini coefficient					
	East		Central		West	
	(1)	(2)	(3)	(4)	(5)	(6)
*z pcharity*	0.011^**^ (2.59)	0.013^***^ (4.32)	0.001 (0.35)	0.001 (0.70)	0.005 (1.24)	0.006^**^ (2.28)
*Lz pcharity*		0.004 (0.80)		0.001 (0.56)		0.002 (0.35)
*Other control variables*	Y	Y	Y	Y	Y	Y
*Province FE*	Y	Y	Y	Y	Y	Y
*Year FE*	Y	Y	Y	Y	Y	Y
*N*	154	161	88	92	198	207
*Adj R^2^*	0.604	0.661	0.713	0.753	0.602	0.645

The *t* values are given in parentheses. ^*^, ^**^, and ^***^ represent significance levels of 10, 5, and 1%, respectively.

Table 5 shows that charitable donations *per capita* widens the urban–rural regional medical level gap. As shown in column (2), an increase of one standard deviation in charitable donations *per capita* increases the urban–rural regional medical level ratio by 0.048, which is significant at the 1% level. That is, the larger the charitable donations *per capita*, the larger the urban–rural regional medical level gap. Although disposable regional medical level *per capita* in the rural area of China is growing, disposable income *per capita* in China’s urban area is growing much more.

The reason for this is that towns and cities have a higher level of economic development, a larger number of high net worth individuals, and a larger number of powerful corporations, thereby increasing the volume of charitable donations. According to the theory of elite philanthropy mentioned above, enterprises and individuals that donate the most are keen to donate, thereby receiving more corresponding returns, which is conducive to their career development and income increase. In rural areas, however, where the level of economic development is relatively lower, and the location is remote, enterprises and residents here donate little or nothing, and their incomes grow slowly or stagnate. Thus, the regional medical level gap between urban and rural areas is getting wider and wider (Table 6).

**Table 6:** Urban–rural heterogeneity.

Variables	Explained variable: urban–rural regional medical ratio	
	(1)	(2)
*z pcharity*	0.033^*^ (1.67)	0.048^***^ (3.23)
*Lz pcharity*		0.024 (1.13)
*Other control variables*	Y	Y
*Province FE*	Y	Y
*Year FE*	Y	Y
*N*	316	316
*Adj R^2^*	0.791	0.790

The *t* values are given in parentheses. ^*^, ^**^, and ^***^ represent significance levels of 10, 5, and 1%, respectively.

## 4 Conclusion and suggestions

Through the empirical analysis of provincial panel data from 1997 to 2019, it is concluded that the current charitable donations *per capita* in China expands the regional medical level gap. And this effect does not only come entirely from the current period but also from the accumulations of past charitable donations. In terms of regional differences, the amount of charitable donations *per capita* expands the regional medical level gap of the east and west regions, and the impact is not significant in the central region. In addition, the charitable donations *per capita* in the eastern region has the greatest expanding effect of the regional medical level gap. In terms of the difference between urban and rural areas, charitable donations *per capita* widens the regional medical level gap between the urban and rural areas, also promotes the increase in the disposable regional medical level *per capita* of the urban areas.

Charitable donations, as the main tool of the tertiary distribution, have slightly widened the regional medical level gap. Although this situation needs to be taken seriously, it is not appropriate to be overly pessimistic. Li (71), the proponent of regional medical level, once pointed out that although the regional medical level currently plays a small role in China’s economy, it is obviously very promising, and its importance will be increasingly recognized in the future. To give better play to the regional medical level of charitable donations and realize common wealth, Chinese government proposed to guide and support the participation of enterprises, social organizations and individuals with the will and ability to participate in public welfare and charitable causes. Specifically, firstly, it is necessary to strengthen charitable publicity, focus on cultivating the charitable awareness of the public, increase the donations of the growing middle regional medical level, get out of the inertia of the elite philanthropy development paths, and optimize the structure of donors while increasing the total amount of donations. Secondly, scientific and reasonable tax incentives for charitable donations need to be designed to stimulate the enthusiasm of enterprises and individuals to make donations, while avoid being captured by high regional medical level, thereby reducing inverted subsidies. Thirdly, strengthen the credibility of charitable organizations, improve the disclosure of information, enhance the transparency of charity, and strengthen supervision and so on to avoid mismatch and inefficiency of the allocation of charitable resources, increasing the trust of residents in charitable activities. Finally, strengthening information sharing, exchange and cooperation between charitable resources and medical assistance, forming policy synergies, and giving full play to the “complementary” role of charitable resources, so that patients can enjoy charitable assistance after enjoying basic medical assistance, thus realizing the rational distribution of limited financial benefits and maximizing the benefits of rehabilitation assistance.

Due to the limitation of data availability, this study did not obtain complete data for 31 provinces, municipalities or autonomous regions across China, which may affect the results. In addition, indicators for measuring the level of charitable development include not only charitable donations and the number of foundations but also volunteer services, charitable trusts, media platforms, and cultural concepts, etc. Different types of charitable giving may have different impacts on regional medical level. And the charitable donations may have different impacts on different types of health care organizations (public hospitals, private hospitals, and public welfare medical organizations). The adjusting effect of charitable donations on income distribution explored in this study may be insufficient to reflect the overall effect, and the possibility that *per capita* charitable giving may have a multi-period impact on regional healthcare levels will be discussed in detail in future research. More importantly, the value and regulating mechanism of charity at the moral and spiritual levels is a subsequent need for further research.

## Data availability statement

The original contributions presented in the study are included in the article/supplementary material, further inquiries can be directed to the corresponding author.

## Author contributions

WS: Conceptualization, Writing – original draft, Writing – review & editing. QZ: Data curation, Formal analysis, Funding acquisition, Writing – original draft, Writing – review & editing.

## Footnotes

^1^In 1997, China received charitable donations totaling140,159,000 yuan, including 415,146,000 yuan in cash and 986,449,000 yuan in donations of clothing and quilts.

^2^Enterprises and other market entities’ donations become a part of the primary distribution by entering the donor’s production and operation cost. The government’s grants, donations, and purchase of social organization services belong to the category of secondary distribution by entering the fiscal budget. As for individual members of society, their voluntary donations belong to the tertiary distribution after obtaining the share of primary and secondary distribution of social products.

^3^Beneficiary groups of social welfare, social assistance and benefits do not have to pay for receiving direct assistance, which is different from the social insurance system. Although their mechanism is essentially similar to that of charity, they are subject to national coordination, which weakens their specificity.

^4^Hebei Economic Yearbook for Hebei Province and Gansu Development Yearbook for Gansu Province.

^5^Sundrum formula cannot effectively solve the situation if the urban and rural income grouping data overlap, which is common in China’s urban and rural income survey data. Although scholars make a lot of explorations for solving this problem, no consensus has been reached. To remain consistent with the data calculated by Zhai et al. (19), this study still chooses the Sundrum formula to calculate the overall urban and rural Gini coefficient after referring to other research practices.

^6^The provinces excluded are Liaoning, Tianjin, Heilongjiang, Anhui, Hunan, Ningxia, Yunnan, and Qinghai.

^7^A rough calculation is conducted to ensure that the sum of provincial donations for the year is close to the national donation volume of the Annual Report on China’s Philanthropy Development.

^8^The number of endowment insurance participants in rural areas has been counted in the China Statistical Yearbook since 2006 (being merged into urban and rural basic endowment insurance since 2010), which is included in the calculation of this study. The working-age population is the population aged 15–64 based on data of age distribution of the population given by each province and the definition of International Labor Organization, of which no direct data are given for 2001. It is estimated based on the population aged 15 or older minus the population aged 65 or older.

^9^GDP for China in 2019 is 9,908.65 billion yuan.

^10^The charitable donations of the United States totaled $449.64 billion in 2019, and GDP was $21.433 trillion.

^11^Due to inconsistence of statistical caliber and data collection, the total amount of charitable donations in China may be seriously underestimated. However, it is indisputable that the amount of charitable donations is not large in China.

^12^There are 19 provinces available in this study. The eastern region includes seven provinces (municipalities): Beijing, Hebei, Shanghai, Jiangsu, Zhejiang, Fujian, and Guangdong. The central region includes four provinces: Shanxi, Jiangxi, Henan, and Hubei. The western region includes eight provinces (autonomous regions): Inner Mongolia, Guangxi, Chongqing, Sichuan, Guizhou, Shaanxi, Gansu, and Xinjiang.
